# Identification and quantification of *Enterococcus faecalis* by real-time quantitative pcr during endodontic treatment

**DOI:** 10.3389/fdmed.2026.1907257

**Published:** 2026-09-09

**Authors:** Shruthi H. Attavar, Rochi Kapoor, Suvarna Kavya Harish, Veena Shetty, Shriya Shetty

**Affiliations:** 1 Nitte (Deemed to be University), AB Shetty Memorial Institute of Dental Sciences (ABSMIDS), Department of Conservative Dentistry and Endodontics, Mangalore, India; 2 Nitte (Deemed to be University), KS Hegde Medical Academy (KSHEMA), Department of Microbiology, Mangalore, India

**Keywords:** biofilm, chlorhexidine, EndoActivator, passive ultrasonic irrigation, refractory endodontic cases, sodium hypochlorite

## Abstract

**Aim:**

To evaluate the quantification of *Enterococcus faecalis* using quantitative polymerase chain reaction (qPCR) and culture techniques following different root canal irrigation protocols in refractory endodontic cases.

**Materials and methods:**

Fifty refractory endodontic cases were randomly allocated into five experimental groups according to the irrigant and activation method used: Group I, saline irrigation; Group II, 5.25% sodium hypochlorite with Passive ultrasonic irrigation; Group III, 5.25% sodium hypochlorite with EndoActivator; Group IV, 2% chlorhexidine with Passive ultrasonic irrigation; and Group V, 2% chlorhexidine with EndoActivator. Microbiological samples were collected before and after disinfection using sterile paper points. Quantification of *Enterococcus faecalis* was performed using conventional culture techniques and qPCR. Data were analyzed using one-way ANOVA, *post hoc* Tukey test, and paired t-tests (*P* < 0.05).

**Results:**

Post-treatment bacterial counts were significantly lower in all experimental groups than in the saline control (*P* < 0.001). Group II (NaOCl with Passive ultrasonic irrigation) showed the greatest reduction, with mean post-treatment counts of 1.73 × 10⁵ CFU/mL by culture and a mean log CFU of 3.56 by qPCR, compared with 408 × 10⁵ CFU/mL and 26.54 log CFU, respectively, in the saline control. Both culture and qPCR demonstrated significant reductions in *E. faecalis* following all active irrigation protocols, with Group II consistently showing the greatest reduction across both detection methods.

**Conclusion:**

5.25% NaOCl activated by Passive ultrasonic irrigation achieved the greatest reduction in *E. faecalis* across both culture and qPCR assessments.

**Clinical significance:**

Passive ultrasonic activation of sodium hypochlorite may enhance root canal disinfection by significantly reducing *Enterococcus faecalis*, a microorganism commonly associated with persistent endodontic infections. The use of qPCR provides a sensitive method for evaluating the antimicrobial efficacy of endodontic irrigation protocols.

## 1 Introduction

 Persistent endodontic infections remain a major challenge in root canal therapy, with *Enterococcus faecalis* being the microorganism most frequently associated with treatment failure and post-treatment apical periodontitis. This facultative Gram-positive anaerobic bacterium has been reported as the predominant species in up to 77% of refractory endodontic cases when identified using conventional culture and polymerase chain reaction (PCR) techniques. The prevalence of *E. faecalis* in secondary endodontic infections ranges from 1.6% to 40% when detected by culture methods and from 7.5% to 14% when identified using conventional PCR techniques (1).

Microbial identification and quantification in endodontic infections are commonly performed using culture-based and molecular techniques. Conventional culture methods are time-consuming, require strict sampling and transportation conditions to maintain microbial viability, and may yield variable results depending on laboratory procedures and operator expertise. In contrast, PCR-based molecular methods offer high sensitivity and specificity for the rapid detection of both cultivable and non-cultivable microorganisms. However, conventional PCR is limited to the qualitative detection of target microorganisms and cannot reliably determine microbial load. Furthermore, it cannot distinguish between viable and non-viable bacteria and requires post-amplification processing for product identification and analysis (2).

Quantitative polymerase chain reaction (qPCR) has emerged as a powerful molecular tool for the detection and quantification of endodontic pathogens. Unlike conventional PCR, qPCR enables real-time monitoring of DNA amplification and allows accurate quantification of microbial populations within clinical samples (3). The technique utilizes calibration curves generated from known concentrations of plasmid DNA, genomic DNA, or other nucleic acid standards to estimate the quantity of target DNA in unknown specimens. This approach facilitates absolute quantification of microorganisms and provides a reliable method for evaluating bacterial load during endodontic treatment. However, a major limitation of conventional qPCR is that it cannot distinguish between DNA derived from viable and non-viable bacterial cells. Consequently, residual DNA from dead microorganisms may still be amplified, potentially leading to an overestimation of bacterial persistence following chemomechanical preparation. To address this limitation, viability-based approaches such as propidium monoazide (PMA)-qPCR have been introduced, allowing preferential amplification of DNA from viable bacterial cells and providing a more accurate assessment of the antimicrobial efficacy of endodontic treatment (4).

Effective root canal disinfection is essential for the success of endodontic therapy. The primary objectives of irrigation are the removal of debris, dissolution of organic tissues, disruption of microbial biofilms, neutralization of endotoxins, and elimination of the smear layer from canal walls. Sodium hypochlorite (NaOCl) is the most widely used endodontic irrigant because of its potent antimicrobial activity, tissue-dissolving capability, and lubricating properties. Chlorhexidine (CHX), a bisbiguanide antiseptic, exhibits broad-spectrum antimicrobial activity and substantivity due to its ability to adsorb onto hydroxyapatite and be released gradually over time. It has demonstrated effectiveness against resistant microorganisms, including *E. faecalis* (5).

Conventional irrigation is typically performed using a needle-and-syringe delivery system. However, this technique has several limitations, particularly in the apical third of the root canal, where irrigant penetration may be inadequate. In addition, irrigant exchange and contact with canal walls are often restricted, reducing its effectiveness in complex root canal anatomies. To overcome these limitations, various irrigant activation systems, including ultrasonic, sonic, and laser-assisted devices, have been introduced to enhance irrigant penetration, fluid dynamics, and antimicrobial efficacy within the root canal system (6).

Several irrigant activation systems have been introduced to enhance the effectiveness of root canal disinfection, including ultrasonic, sonic, and laser-assisted techniques. In the present study, Passive ultrasonic irrigation (PUI) and the EndoActivator were selected because they are among the most widely investigated and clinically adopted activation systems. PUI has consistently demonstrated superior acoustic streaming and cavitation effects that improve irrigant penetration and removal of debris and microorganisms from the root canal system. The EndoActivator, a sonic activation device, is widely used due to its simplicity, safety, and ability to enhance irrigant circulation without direct contact with canal walls. A comparison of these two established activation methods provides clinically relevant evidence regarding their antimicrobial efficacy while minimizing the influence of differences associated with newer technologies, such as laser-assisted irrigation, which require specialized equipment and operate through different mechanisms.

Quantitative PCR offers significant advantages over conventional PCR and culture-based methods by providing rapid, highly sensitive, and species-specific quantification of *Enterococcus faecalis*. Unlike conventional PCR, which only confirms the presence of bacterial DNA, qPCR measures bacterial load, enabling accurate assessment of antimicrobial efficacy. Compared with culture methods, qPCR detects bacteria that are present in low numbers or in a viable but non-culturable state, making it particularly valuable for evaluating persistent endodontic infections (4). Therefore, accurate quantification of microbial reduction following different irrigation protocols is essential for evaluating the effectiveness of endodontic disinfection procedures. The present study aimed to compare the quantification of *Enterococcus faecalis* using conventional culture methods and quantitative polymerase chain reaction following different irrigation protocols in refractory endodontic cases.

Null hypothesis: There is no significant difference in the microbial reduction following different irrigation protocols when detected using culture and qPCR technique.

## 2 Material and methods

### 2.1 Ethical approval

The study protocol was approved by the Institutional Ethics Committee (Approval No. ABSM/EC/124/2021) and conducted in accordance with the Declaration of Helsinki (1975, revised 2013). Written informed consent was obtained from all participants prior to enrollment; for participants below 18 years of age, written informed consent was obtained from a parent or legal guardian, in addition to assent from the minor participant. Fifty patients aged 15–45 years who met the eligibility criteria were recruited for the study.

### 2.2 Sample size calculation

Sample size estimation was performed using nMaster software (version 2.0) based on pilot study data. Considering a pooled standard deviation of 0.523, an expected mean difference of 0.61, an effect size of 1.16, a significance level of 5%, and a study power of 80%, a minimum sample size of 10 specimens per group was required, resulting in a total sample size of 50 (7).

### 2.3 Inclusion criteria

The following teeth were included:

Previously root canal-treated teeth with persistent periapical radiolucency suggestive of post-treatment apical periodontitis.

Root canal treatment performed at least 5 years prior to presentation.

Root canal filling terminating within 5 mm of the radiographic apex.

Adequate coronal tooth structure to permit rubber dam isolation.

### 2.4 Exclusion criteria

Teeth presenting with periodontal pockets >4 mm, dystrophic calcification, acute apical abscess, root perforation, external or internal resorption, immature apices, or patients who had received systemic antibiotics within the previous 3 months were excluded. Pregnant women and medically compromised patients were also excluded.

### 2.5 Clinical protocol

#### 2.5.1 Randomization, allocation concealment, and blinding

Patients were randomly allocated to the five treatment groups using a computer-generated random number sequence, with allocation concealed using sequentially numbered, sealed opaque envelopes opened only after eligibility was confirmed. Owing to the visibly distinct irrigants and devices used, the operator could not be blinded to group allocation; however, personnel performing bacterial quantification and statistical analysis were blinded using coded sample identifiers. Baseline pretreatment bacterial counts were comparable among groups for both culture and qPCR (*P* = 0.599 and *P* = 0.147, respectively; Table 1).

**Table 1:** One-way ANOVA showing the Microbial load before and after treatment with irrigating devices (culture method and Real-time PCR).

Bacterial load quantification	Groups	N	Mean	Std. Deviation	Statistic/F	*P* value
CFU before treatment (in 10^5)	Group 1	10	408	170.4112672	0.696	0.599
	Group 2	10	361	154.7363349		
	Group 3	10	427	126.9339286		
	Group 4	10	454	115.6815552		
	Group 5	10	372	154.113955		
CFU After treatment (in 10^5)	Group 1	10	408	170.4112672	10.077	.000*
	Group 2	10	152.3	46.46635580		
	Group 3	10	184.10	89.2953277364		
	Group 4	10	211.00	80.410060		
	Group 5	10	166.900	94.670715		
	Total	50	224.46	96.250712		
PCR (Log CFU) pre treatment	Group 1	10	1.5262	0.318947	4.111	.006
	Group 2	10	1.236	0.353999		
	Group 3	10	1.987	0.474342		
	Group 4	10	1.718	0.396955		
	Group 5	10	1.604	0.561074		
	Total	50	1.61424	0.47879		
PCR (Log CFU) post treatment	Group 1	10	1.107	0.284412	11.131	.000*
	Group 2	10	0.363	0.293789		
	Group 3	10	0.86	0.238654		
	Group 4	10	0.52	0.291204		
	Group 5	10	0.629	0.274406		
	Total	50	0.6958	0.374711		

*Statistically significant.

#### 2.5.2 Sample collection

Both single-rooted and multirooted teeth were included, with tooth type distributed comparably across the five groups. In multirooted teeth, only the canal associated with the largest periapical radiolucency was sampled and included in analysis.

The selected tooth was isolated using a rubber dam prior to the retreatment procedure. Working length was determined using an apex locator (Root ZX, J. Morita) and confirmed radiographically, set 1 mm short of the radiographic apex. Existing gutta-percha was removed using the ProTaper Universal Retreatment file system up to file D3 (Dentsply Maillefer, Ballaigues, Switzerland). No root canal solvent was used during gutta-percha retrieval. Saline was used as the irrigant during the shaping phase for all groups, ensuring that chemomechanical preparation did not introduce any antimicrobial effect prior to randomization and application of the experimental irrigation protocol.

For the initial microbiological sampling, a sterile paper point was inserted into the root canal to the working length, approximately 1 mm short of the apical foramen, and maintained in position for 60 s. Prior to insertion of the paper point, the canal was dried using sterile absorbent paper points to remove residual irrigant or debris. In multirooted teeth, the sample was obtained from the canal associated with the largest periapical radiolucency. The paper point was subsequently transferred to a sterile Eppendorf tube containing 200 μL of Dulbecco's phosphate-buffered saline (HiMedia Laboratories, India) for bacterial quantification using culture and quantitative polymerase chain reaction (qPCR) analysis (8).

Following sample collection, chemomechanical preparation was performed using the ProTaper Gold rotary file system (Dentsply Maillefer, Ballaigues, Switzerland) up to size 30. The teeth were then randomly assigned to one of the following irrigation protocols:

- Group I (*n* = 10): Saline irrigation (control group).
- Group II (*n* = 10): 5.25% sodium hypochlorite (Parcan N, Septodont, UK) followed by Passive ultrasonic irrigation (Irrisafe, Satelec Acteon, France) for 60 s.
- Group III (*n* = 10): 5.25% sodium hypochlorite followed by sonic activation using the EndoActivator system (Dentsply Sirona, USA) for 60 s.
- Group IV (*n* = 10): 2% chlorhexidine (Asep-RC, Stedman Pharmaceuticals Pvt. Ltd., India) followed by Passive ultrasonic irrigation for 60 s.
- Group V (*n* = 10): 2% chlorhexidine followed by sonic activation using the EndoActivator system for 60 s.

Irrigation was performed using a total volume of 5 mL per canal, delivered via a 30-gauge side-vented needle placed 2–3 mm short of working length. Following the respective irrigation protocol, all canals received a final rinse with 17% EDTA for 60 s to remove the smear layer, followed by a saline rinse (9).

Post-treatment sampling was performed immediately following completion of the irrigation protocol, with a total elapsed time of approximately 15 min between pre- and post-treatment sample collection. No intracanal medicament was placed between sampling time points, as both samples were obtained within a single visit.

Following irrigation, a second microbiological sample was collected using the same sampling protocol. A sterile paper point was placed at the working length, approximately 1 mm short of the apical foramen, for 60 s and then transferred to an Eppendorf tube containing 200 μL of Dulbecco's phosphate-buffered saline. These samples were subsequently subjected to culture-based analysis and qPCR for the quantification of bacterial load.

#### 2.5.3 Experimental procedure 1: quantification by culture

Microbiological samples were subjected to ten-fold serial dilution in sterile saline solution. Subsequently, 100 μL aliquots from the appropriate dilutions were inoculated onto Brain Heart Infusion (BHI) agar plates and incubated at 37 °C for 48 h. Following incubation, the resulting colonies were counted and expressed as colony-forming units per milliliter (CFU/mL). The bacterial counts were calculated by considering the number of colonies obtained and the corresponding dilution factor (10).

The CFU/mL was calculated using the following formula:

### 2.6 CFU/mL = (number of colonies   ×   dilution factor)/volume plated (mL)

#### 2.6.1 Experimental procedure 2: DNA extraction and real-time PCR

Following the extraction, processing, and normalization of microbe genome DNA using the All Prep DNA/RNA/miRNA Universal Kit (Takara, Otsu, Shiga, Japan), the final DNA concentration was confirmed using a NanoDrop Spectrophotometer. 19 μL reaction mixtures consisting of extracted DNA, sterile water, a particular primer-probe combination, and universal PCR Master Mix were used for qPCR. A 96-well plating system was used for the 40-cycle PCR, which was run using Bio-Rad CFX Maestro Software, which included denaturation, annealing, and extension. Before and after irrigation with various solutions, observations of the bacterial loading and cycle threshold (Ct) value were tallied in Excel for analysis (11).

Quantitative PCR was performed using a TaqMan assay targeting the *Enterococcus faecalis* 16S rRNA gene. The forward primer sequence was 5′ CTTATGACCTGGGCTACACAC-3′, the reverse primer sequence was 5′-CTGCAATCCGAACTGAGAGAA-3′, and the hydrolysis probe sequence was 5′-Cy5-ACCGCGAGGTCATGCAAATCTCTT-3′ with an internal TAO quencher and a 3′ Iowa Black RQ-Sp quencher, generating a 103-bp amplicon.

No viability-based PCR approach, such as propidium monoazide qPCR (PMA-qPCR), was employed in this study; qPCR therefore quantified total bacterial DNA without distinguishing between viable and non-viable cells. All samples were analyzed in duplicate, and the mean Ct value across replicates was used for quantification. Bacterial load was expressed per sample volume without normalization to total DNA concentration, with all samples processed from a fixed elution volume of 200 μL phosphate-buffered saline. The limit of detection of the assay, determined from the lowest standard dilution yielding a reproducible signal, was 1.0 log₁₀ CFU/mL.

#### 2.6.2 Conversion of Ct values to log CFU

A standard curve was generated using 10-fold serial dilutions of an *Enterococcus faecalis* culture with known viable counts, determined by conventional plate counting and expressed as colony-forming units per milliliter (CFU/mL). Genomic DNA was extracted from each dilution and analyzed by quantitative PCR under identical amplification conditions. The Ct values obtained were plotted against the corresponding log_10_ CFU/mL to generate a linear regression equation.

The relationship between Ct and bacterial concentration was described by the equation:

Ct = m(\log_{10}CFU) + b

where *m* is the slope and *b* is the y-intercept of the standard curve.

For each unknown sample, the Ct value was substituted into the regression equation and rearranged to calculate the bacterial concentration:

[\log_{10}(\mathrm{CFU}/\mathrm{mL})=\frac{\mathrm{Ct}-b}{m}]

The calculated log CFU values were corrected for any dilution factors and expressed as log_10_ CFU/mL (or log_10_ CFU per sample, as appropriate). Only standard curves with a correlation coefficient (R^2^) ≥ 0.99 and amplification efficiencies between 90% and 110% were accepted for quantitative analysis.

### 2.7 Statistical analysis

Descriptive statistics were calculated, and box plots were generated to summarize and visually compare the distribution of bacterial counts among the study groups. Continuous variables were expressed as mean, standard deviation, and 95% confidence intervals.

Prior to analysis, normality was assessed using the Shapiro–Wilk test and homogeneity of variance was evaluated using Levene's test. While normality was confirmed for the majority of subgroups, departures were observed in Group 3 for CFU data (*P* = 0.046 before treatment; *P* = 0.001 after treatment) and in Groups 1 and 2 for PCR post-treatment data (*P* = 0.008 and *P* = 0.017, respectively). Given the balanced design with equal group sizes across all five groups, one-way ANOVA and paired t-tests were retained for the primary analyses, as these tests are considered robust to mild violations of normality under such conditions (Table 2).

**Table 2:** Tests for normality.

Variable	Group	Shapiro–Wilk		
		Statistic	df	Sig.
CFU before treatment (in 10^5)	1	.954	10	.714
	2	.945	10	.607
	3	.841	10	.046
	4	.947	10	.627
	5	.969	10	.881
CFU after treatment (in 10^5)	1	.954	10	.714
	2	.894	10	.188
	3	.695	10	.001
	4	.952	10	.690
	5	.850	10	.058
PCR (Log CFU) pre treatment	1	.729	10	.002
	2	.935	10	.502
	3	.944	10	.595
	4	.970	10	.895
	5	.863	10	.084
PCR (Log CFU) post treatment	1	.779	10	.008
	2	.805	10	.017
	3	.977	10	.946
	4	.940	10	.551
	5	.913	10	.302

One-way analysis of variance (ANOVA) was used to evaluate differences in bacterial reduction among the five experimental groups. When significant differences were identified, *post hoc* pairwise comparisons were performed using Tukey's multiple comparison test.

A paired t-test was used to compare bacterial counts before and after treatment within each group.

The statistical approach was designed to answer two separate questions: whether the irrigation protocols differed from one another in their effect on bacterial load, and whether each protocol actually reduced bacterial counts within the treated teeth. One-way ANOVA with *post hoc* Tukey testing addressed the first question by comparing groups at each time point, while paired t-tests addressed the second by comparing pre- and post-treatment values within each group. A repeated-measures ANOVA was considered but was not applied, as the study was not designed to model change-over time but rather to capture a single, discrete treatment effect within a single visit.

Statistical analyses were performed at a significance level of 5%, and a *P* value < 0.05 was considered statistically significant.

## 3 Results

The present study compared bacterial quantification using conventional culture and quantitative polymerase chain reaction (qPCR) before and after disinfection with different irrigation protocols. Bacterial counts were expressed as colony-forming units (CFU) for culture analysis and log CFU values for qPCR analysis.

One-way analysis of variance (ANOVA) was used to evaluate differences in bacterial counts among the five experimental groups (Table 1). For culture-based analysis, no statistically significant difference was observed in pretreatment CFU counts among the groups (*P* = 0.599). However, significant differences were observed in post-treatment CFU counts, with Group II (5.25% sodium hypochlorite with Passive ultrasonic irrigation) demonstrating the lowest bacterial counts and Group I (saline control) exhibiting the highest counts.

Similarly, qPCR analysis revealed a significant difference in pretreatment bacterial counts among the groups (*P* = .006) In contrast, post-treatment log CFU values differed significantly among the groups, with Group II demonstrating the greatest reduction in bacterial load and Group I exhibiting the least reduction.

Post hoc multiple comparison analysis (Table 3) demonstrated no significant differences among the groups at baseline for either culture or qPCR measurements. Following treatment, culture-based analysis revealed significantly lower CFU counts in Groups II, III, IV, and V compared with the control group (Group I) (*P* < 0.001). Likewise, qPCR analysis demonstrated significant differences between Group I and Groups III, IV, and V (*P* < 0.001). Significant differences were also observed between Group II and Group III, and between Group II and Group V (*P* < 0.001).

**Table 3:** *Post hoc* tukey test showing the multiple comparison within different groups (culture + real-time PCR).

Variable	Comparison of	Comparison with	Mean difference	Standard error	*P* value
CFU before treatment (in 10^5)	Group 1	Group 2	47	65.18555	0.7530
		Group 3	−19	65.18555	0.9980
		Group 4	−46	65.18555	0.9540
		Group 5	36	65.18555	0.9810
	Group 2	Group 3	−66	65.18555	0.8480
		Group 4	−93	65.18555	0.6140
		Group 5	−11	65.18555	1.0000
	Group 3	Group 4	−27	65.18555	0.9940
		Group 5	55	65.18555	0.9150
	Group 4	Group 5	82	65.18555	0.7180
CFU after treatment (in 10^5)	Group 1	Group 2	255.7000000*	46.73375	.000*
		Group 3	223.9000000*	46.73375	.000*
		Group 4	197.0000000*	46.73375	.001*
		Group 5	241.0000000*	46.73375	.000*
	Group 2	Group 3	31.799999999	46.73375	.960
		Group 4	58.699999999	46.73375	.719
		Group 5	14.599999999	46.73375	.998
	Group 3	Group 4	26.900000000	46.73375	.978
		Group 5	17.199999999	46.73375	.996
	Group 4	Group 5	44.099999999	46.73375	.878
PCR (Log CFU) pre treatment	Group 1	Group 2	0.2902	.191211	.557
		Group 3	-.460800	.191211	.131
		Group 4	−0.191800	.191211	.853
		Group 5	−0.077800	.191211	.994
	Group 2	Group 3	−0.75100	.191211	.003*
		Group 4	−0.48200	.191211	.104
		Group 5	−0.36800	.191211	.320
	Group 3	Group 4	0.269000	.191211	.627
		Group 5	0.383000	.191211	.281
	Group 4	Group 5	0.114000	.191211	.975
PCR (Log CFU) post treatment	Group 1	Group 2	0.74400	.12398	.000*
		Group 3	0.24700	.12398	.286
		Group 4	0.58700	.12398	.000*
		Group 5	0.47800	.12398	.003*
	Group 2	Group 3	−0.49700	.12398	.002*
		Group 4	−0.15700	.12398	.713
		Group 5	−0.26600	.12398	.219
	Group 3	Group 4	O.34000	.12398	.063
		Group 5	0.23100	.12398	.352
	Group 4	Group 5	−0.10900	.12398	.903

*Statistically significant.

*The mean difference is significant at the 0.05 level.

Within-group comparisons using the paired t-test demonstrated a significant reduction in bacterial counts following irrigation in all experimental groups, irrespective of the detection method employed. Both culture and qPCR analyses showed significant reductions in bacterial load after treatment compared with baseline values (*P* < 0.001) (Table 4).

**Table 4:** Paired t test showing the microbial analysis before and after treatment with irrigating devices (culture + real-time PCR).

Group	Quantification method	Variable	N	Mean ± SD	Mean difference ± SD	t	*P* value
Group 1	Culture method	CFU Before Treatment (in 10^5)	10	408 ± 170.41			
		CFU After Treatment (in 10^5)	10	408 ± 170.41			
	qPCR	PCR (Log CFU) Pre Treatment	10	1.5262 ± 0.318947	.419200 ± 40783	3.250	010*
		PCR (Log CFU) Post Treatment	10	1.107 ± 0.284112			
Group 2	Culture method	CFU Before Treatment (in 10^5)	10	361 ± 154.74	208.700 ± 140.502	4.697	.001*
		CFU After Treatment (in 10^5)	10	152.3 ± 46.46355			
	qPCR	PCR (Log CFU) Pre Treatment	10	1.236 ± 0.353999	.8730 ± .78517	5.769	.000*
		PCR (Log CFU) Post Treatment	10	0.363 ± 0.293789			
Group 3	Culture method	CFU Before Treatment (in 10^5)	10	427 ± 126.93	242.9 ± 68.885	11.151	.000*
		CFU After Treatment (in 10^5)	10	184.10 ± 89.2953			
	qPCR	PCR (Log CFU) Pre Treatment	10	1.987 ± 0.474342	1.127 ± 0.235688	6.900	.000*
		PCR (Log CFU) Post Treatment	10	1.127 ± .516506			
Group 4	Culture method	CFU Before Treatment (in 10^5)	10	454 ± 115.68	243.00 ± 93.578	8.212	.000*
		CFU After Treatment (in 10^5)	10	211.00 ± 80.41006			
	qPCR	PCR (Log CFU) Pre Treatment	10	1.718 ± 0.396955	1.198 ± .561818	6.743	.000*
		PCR (Log CFU) Post Treatment	10	0.52 ± 0.291204			
Group 5	Culture method	CFU Before Treatment (in 10^5)	10	372 ± 154.11	205.10 ± 134.25	4.831	.001*
		CFU After Treatment (in 10^5)	10	166.900 ± 94.6707			
	qPCR	PCR (Log CFU) Pre Treatment	10	1.604 ± 0.561074	0.975 ± .713322	4.322	.002*
		PCR (Log CFU) Post Treatment	10	0.629 ± 0.274406			

*Statistically significant.

## 4 Discussion

Successful endodontic treatment depends on effective disinfection of the root canal system. Although mechanical instrumentation and irrigation significantly reduce the microbial burden, complete elimination of microorganisms from the complex root canal anatomy remains challenging. Microorganisms can persist within dentinal tubules and inaccessible areas of the canal system, where they may survive and proliferate within protective biofilms. Therefore, the final irrigation protocol plays a critical role in achieving optimal canal disinfection and improving treatment outcomes (12).

The ability of *Enterococcus faecalis* to penetrate dentinal tubules and survive under adverse environmental conditions contributes to its frequent association with persistent and secondary endodontic infections. The presence of a smear layer may further influence microbial colonization and limit the penetration of antimicrobial agents into dentinal tubules. Consequently, effective smear layer removal is essential for improving root canal disinfection and reducing bacterial persistence (13).

In the present study, 5.25% sodium hypochlorite was selected as an irrigant because previous investigations have demonstrated that higher concentrations of sodium hypochlorite are more effective in eliminating *E. faecalis* from dentinal tubules, although different concentrations may exhibit comparable antibacterial effects within the root canal lumen (14). Similarly, 2% chlorhexidine was selected because of its broad-spectrum antimicrobial activity and substantivity. At higher concentrations, chlorhexidine exhibits bactericidal activity, whereas lower concentrations primarily exert a bacteriostatic effect.

Passive ultrasonic irrigation and sonic activation using the EndoActivator were employed as final irrigation activation techniques. These systems enhance irrigant penetration and distribution within the root canal system by generating hydrodynamic movement and improving fluid exchange. The oscillatory motion produced by these devices promotes debris removal and smear layer disruption, thereby facilitating improved contact between the irrigant and the canal walls (15).

Previous studies investigating *E. faecalis* in endodontic infections have primarily relied on culture methods or conventional PCR for microbial identification. The present study evaluated the usefulness of a molecular-based approach for the detection and quantification of *E. faecalis* in infected root canals. Compared with cultivation-based investigations, relatively few studies have assessed the prevalence and quantification of *E. faecalis* using advanced molecular techniques in refractory endodontic infections (16).

In the present study both culture and qPCR were employed as parallel quantification tools to assess the effect of different irrigation protocols on *E. faecalis* load. Various studies have demonstrated qPCR to be more sensitive than conventional culture methods for detecting and quantifying bacterial load. However, although molecular methods provide enhanced sensitivity and specificity, they also possess certain limitations. qPCR detects bacterial DNA irrespective of cell viability and therefore cannot distinguish between viable and non-viable microorganisms. In addition, DNA extraction procedures prevent subsequent cultivation and phenotypic characterization of bacterial strains. Consequently, molecular and culture-based methods should be considered complementary approaches, as their combined use provides a more comprehensive understanding of the microbial profile associated with endodontic infections (17).

The present study also demonstrated that 5.25% sodium hypochlorite exhibited greater antimicrobial efficacy against *E. faecalis* than 2% chlorhexidine. This finding is in agreement with previous reports that have attributed the superior antibacterial activity of sodium hypochlorite to its potent antibiofilm action and tissue-dissolving capability. By disrupting bacterial adhesion and facilitating the removal of microorganisms embedded within biofilms, sodium hypochlorite contributes to more effective root canal disinfection (18).

The null hypothesis was partially accepted. All activated irrigation protocols demonstrated significantly greater bacterial reduction than the control group when assessed using both culture and qPCR techniques. However, no statistically significant differences were observed among sodium hypochlorite activated with EndoActivator, chlorhexidine activated with EndoActivator, and chlorhexidine activated with Passive ultrasonic irrigation, indicating comparable antimicrobial effectiveness among these protocols (19).

Among all experimental groups, Group II, comprising 5.25% sodium hypochlorite activated with Passive ultrasonic irrigation, demonstrated the greatest reduction in bacterial load. This enhanced antimicrobial effect may be attributed to the high-frequency ultrasonic oscillations that generate acoustic streaming and cavitation within the irrigating solution. These phenomena increase shear stress along canal walls, disrupt bacterial biofilms, and improve irrigant penetration into inaccessible areas of the root canal system. Furthermore, ultrasonic activation improves fluid dynamics and may enhance the antibacterial and cleaning properties of the irrigating solution. The effectiveness of ultrasonic irrigation is influenced by several factors, including instrument size, taper, tip diameter, canal anatomy, and the type of irrigant used (20).

The superior performance of sodium hypochlorite observed in the present study may also be attributed to its unique chemical properties. Upon contact with organic tissues, sodium hypochlorite forms hypochlorous acid, which disrupts bacterial metabolism through oxidation of essential cellular components, ultimately resulting in bacterial cell death. Furthermore, passive ultrasonic activation enhances the tissue-dissolving and antimicrobial properties of sodium hypochlorite by improving irrigant penetration and contact with canal walls, thereby facilitating more effective removal of organic and inorganic debris from the root canal system (21).

### 4.1 Limitations

The present study evaluated the antimicrobial efficacy of different irrigation protocols against only one microorganism, *Enterococcus faecalis.* Endodontic infections are polymicrobial in nature, and future investigations should assess the effects of these irrigation protocols on a broader spectrum of endodontic pathogens. With only 10 teeth per group, the sample size was relatively small, which may have limited the statistical power of the comparisons and restricts how broadly these findings can be generalized. Larger, multicenter trials would help confirm whether the observed differences hold across a wider patient population. Differences in canal anatomy and the complexity of each retreatment case, despite standardized inclusion criteria, may also have introduced some variability in sampling and outcomes.

The operator could not be blinded to group allocation, since the irrigants and activation devices were visibly distinct, which may have introduced a degree of performance bias. Because qPCR detects bacterial DNA regardless of cell viability, it cannot distinguish between viable and non-viable *Enterococcus faecalis*, and this may have led to an overestimation of residual bacterial load after treatment. The microbiological endpoint assessed here was also limited to an immediate, short-term measurement, with no clinical or radiographic follow-up; how this immediate bacterial reduction translates into long-term healing of periapical lesions therefore remains unclear. Finally, paper point sampling, although widely used, may not fully capture bacteria sequestered within dentinal tubules, isthmuses, or biofilm structures, which could mean the true residual microbial burden was underestimated. In addition, larger clinical studies incorporating molecular analyses of the complete endodontic microbiome may provide a more comprehensive assessment of disinfection efficacy.

## 5 Conclusion

Within the limitations of the present study, both culture and qPCR demonstrated significant reductions in *E. faecalis* load following all active irrigation protocols. Among the irrigation protocols evaluated, 5.25% sodium hypochlorite activated using Passive ultrasonic irrigation demonstrated the greatest reduction in the *E. faecalis* count.

### 5.1 Clinical significance

Effective root canal disinfection is essential for the long-term success of endodontic treatment. The findings of the present study suggest that passive ultrasonic activation of sodium hypochlorite enhances bacterial reduction within the root canal system compared with conventional irrigation protocols. Improved microbial elimination may contribute to more effective canal disinfection, thereby potentially reducing the risk of persistent infection, reinfection, and subsequent endodontic treatment failure. However, further well-designed clinical studies with long-term follow-up are required to confirm whether the observed microbiological benefits translate into improved treatment outcomes.

## Data Availability

The original contributions presented in the study are included in the article/Supplementary Material, further inquiries can be directed to the corresponding author.
